# *N*,*N*,*N*,*N*′,*N*′,*N*′-Hexa­kis­(2-hy­droxy­ethyl)butane-1,4-diaminium bis­(2-sul­fan­ylidene-1,3-di­thiole-4,5-dithiolato-κ^2^ *S* ^4^,*S* ^5^)zincate

**DOI:** 10.1107/S1600536813014992

**Published:** 2013-06-08

**Authors:** Xiulan Zhang, Bin Xie, Like Zou, Jun Wang, Xiao Lin

**Affiliations:** a Institute of Functionalized Materials, Sichuan University of Science and Engineering, Zigong 643000, People’s Republic of China; b College of Chemistry and Pharmaceutical Engineering, Sichuan University of Science and Engineering, Zigong 643000, People’s Republic of China

## Abstract

In the asymmetric unit of the title compound, (C_16_H_38_N_2_O_6_)[Zn(C_3_S_5_)_2_], two independent cations lie across inversion centers. In one of the cations, the three symmetry-unique O—H groups are disordered over two sets of sites with refined occupancy ratios of 0.701 (9):0.299 (9), 0.671 (8):0.329 (8) and 0.566 (7):0.434 (7). In the anion, the Zn^II^ ion is coordinated in a distorted tetra­hedral environment by four S atoms of two chelating 1,3-di­thiole-2-thione-4,5-dithiolato ligands. The dihedral angle between the mean planes [maximun deviations = 0.022 (3) and 0.0656 (6) Å] of the two ligands is 87.76 (3)°. An intamolecular O—H⋯O hydrogen bond occurs in the disordered cation. In the crystal, O—H⋯O and O—H⋯S hydrogen bonds link the components into a two-dimensional network parallel to (0-11).

## Related literature

For synthetic background to the title compound, see: Steimecke *et al.* (1982 ▶); Xie *et al.* (2009 ▶). For a related crystal structure, see: Zhao *et al.* (2011 ▶).
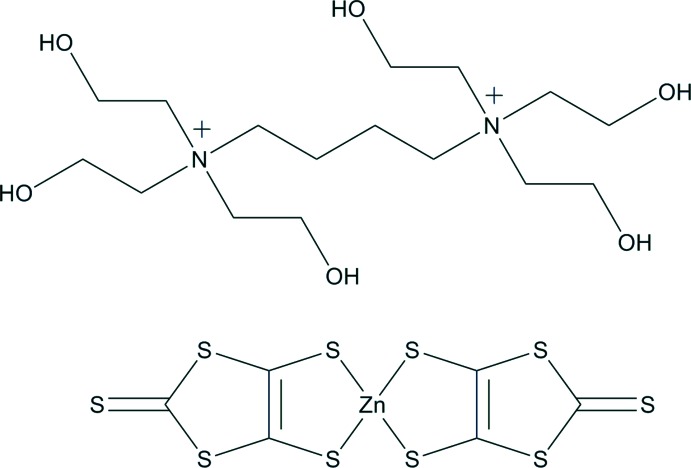


## Experimental

### 

#### Crystal data

(C_16_H_38_N_2_O_6_)[Zn(C_3_S_5_)_2_]
*M* *_r_* = 812.51
Triclinic, 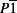

*a* = 9.051 (5) Å
*b* = 13.142 (7) Å
*c* = 15.321 (8) Å
α = 69.803 (5)°
β = 84.566 (5)°
γ = 76.909 (6)°
*V* = 1665.6 (15) Å^3^
*Z* = 2
Mo *K*α radiation
μ = 1.40 mm^−1^
*T* = 296 K
0.21 × 0.20 × 0.20 mm

#### Data collection

Bruker SMART APEXII CCD diffractometer
Absorption correction: multi-scan (*SADABS*; Bruker, 2008 ▶) *T*_min_ = 0.757, *T*_max_ = 0.767
11410 measured reflections
7406 independent reflections
6279 reflections with *I* > 2σ(*I*)
*R*_int_ = 0.017

#### Refinement

*R*[*F* ^2^ > 2σ(*F* ^2^)] = 0.035
*wR*(*F* ^2^) = 0.098
*S* = 1.05
7406 reflections
401 parameters
4 restraints
H-atom parameters constrained
Δρ_max_ = 0.89 e Å^−3^
Δρ_min_ = −0.61 e Å^−3^

### 

Data collection: *APEX2* (Bruker, 2008 ▶); cell refinement: *SAINT* (Bruker, 2008 ▶); data reduction: *SAINT*; program(s) used to solve structure: *SHELXS97* (Sheldrick, 2008 ▶); program(s) used to refine structure: *SHELXL2013* (Sheldrick, 2008 ▶); molecular graphics: *PLATON* (Spek, 2009 ▶) and *Mercury* (Macrae *et al.*, 2008 ▶); software used to prepare material for publication: *SHELXL2013*.

## Supplementary Material

Crystal structure: contains datablock(s) global, I. DOI: 10.1107/S1600536813014992/lh5618sup1.cif

Structure factors: contains datablock(s) I. DOI: 10.1107/S1600536813014992/lh5618Isup2.hkl

Additional supplementary materials:  crystallographic information; 3D view; checkCIF report

## Figures and Tables

**Table 1:** Hydrogen-bond geometry (Å, °)

*D*—H⋯*A*	*D*—H	H⋯*A*	*D*⋯*A*	*D*—H⋯*A*
O1—H1⋯O5^i^	0.82	2.03	2.800 (5)	157
O2—H2⋯O5^i^	0.82	1.93	2.668 (5)	149
O3—H3⋯O2^ii^	0.82	2.16	2.923 (3)	154
O4—H4⋯O6^i^	0.82	2.07	2.887 (8)	179
O5—H5⋯O6	0.82	1.79	2.615 (9)	179
O6—H6⋯S2^iii^	0.82	2.92	3.540 (8)	134

Symmetry codes: (i) 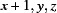; (ii) 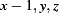; (iii) 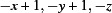.

**Fig. 1.**
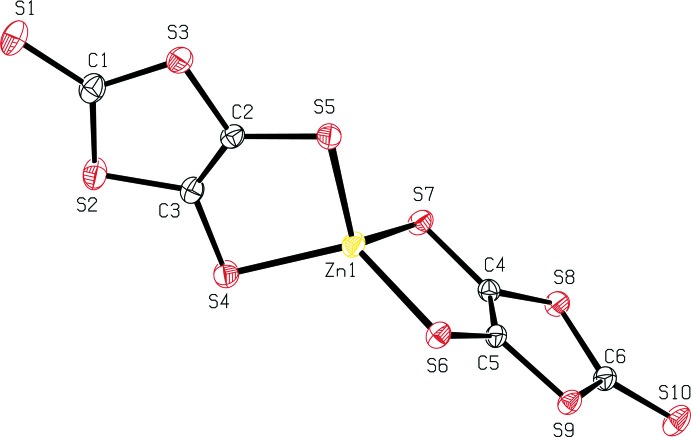
The anion with displacement ellipsoids shown at the 30% level.

**Fig. 2.**
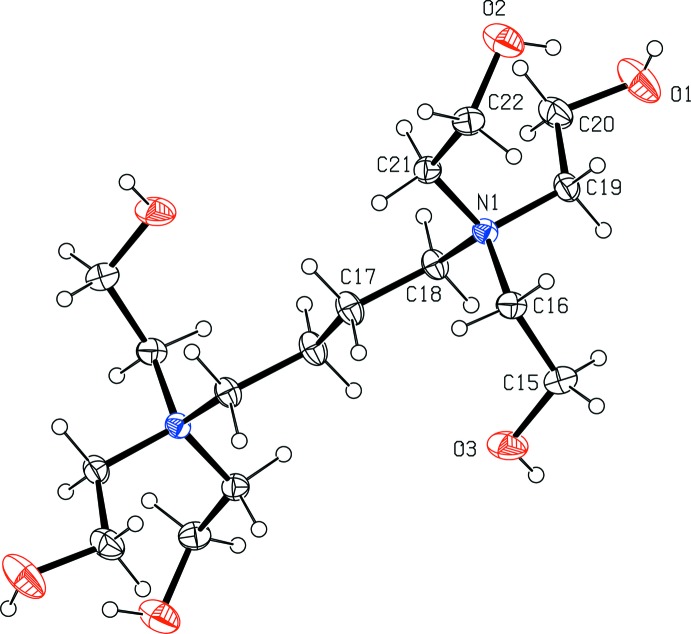
The ordered cation with displacement ellipsoids shown at the 30% level. Unlabled atoms are related by the symmetry operator (-x + 1, -y + 2, -z + 1).

**Fig. 3.**
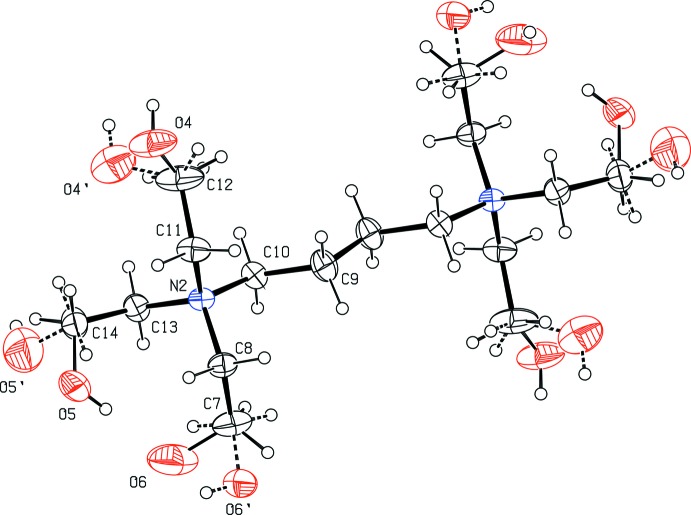
The disordered cation with displacement ellipsoids shown at the 30% level. Unlabled atoms are related by the symmetry operator (-x + 1, -y + 1, -z). The minor components are shown with dashed lines.

**Fig. 4.**
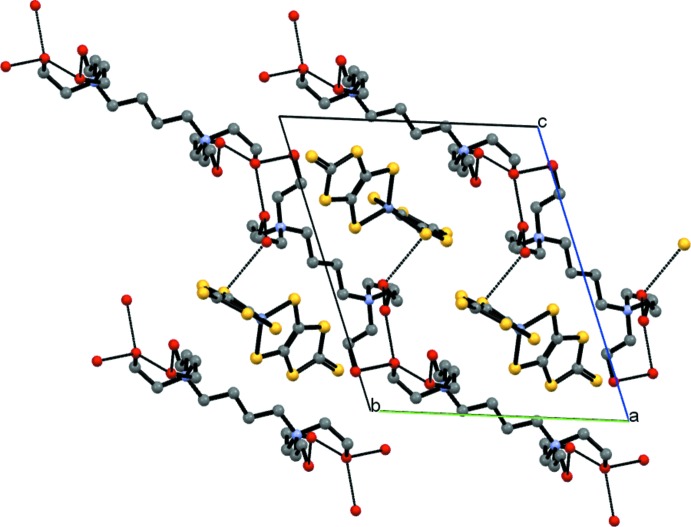
Part of the crystal structure with donor-acceptor distances of hydrogen bonds shown as dashed lines.

## supplementary crystallographic information

### Comment

The crystal structure of the title compound is presented herein. The anion is shown in Fig. 1 and the cations are shown in Figs. 3 and 4. The asymmetric unit contains two independent cations which lie across inversion centers. In one of the cations the three symmetry unique O—H groups are disordered over two sets of sites with refined occupancy ratios of 0.701 (9):0.299 (9) for O4:O4', 0.671 (8):0.329 (8) for O5:O5' and 0.567 (7):0.434 (7) for O6:O6'. In the anion, the Zn^II^ ion is coordinated in a distorted tetrahedral coordination environment by four S atoms of two chelating 1,3-dithiole-2-thione-4,5-dithilato ligands. The dihedral angle between the mean planes of the two ligands (with maximun deviations of 0.022 (3) for C3 and 0.0656 (6) Å for S8) is 87.76 (3)°. In the crystal, O—H···O and O—H···S hydrogen bonds link the components of the structure into a two-dimensional network parallel to (011) (Fig. 4). Only the donor H atoms of the major components of disorder have been considered. The bis(tetra-*N*-butylammoniun) analogue of the title compound has been reported in the literature (Zhao *et al.*, 2011).

### Experimental

All synthetic procedures were carried out under nitrogen using standard Schlenk techniques. The compound tdb(COPh)_2_ (tdb = 4,5-dimercapto-1,3-dithiole-2-thione) was synthesized according to procedure described by Steimecke *et al.* (1982) and Xie *et al.* (2009). The quatemary ammonium salt *N*,*N*'-bis(tri(2'-hydroethyl)-1,4-butanediammonium bromide [BDA]Br_2_ was obtained by the reaction of triethanolamine (30 g, 0.2 mol) and 1,4-dibromobutane (21 g, 0.1 mol) in refluxing acetone for one week. The compound tdb(COPh)_2_ (0.812 g, 2 mmol) was dissolved in sodium methoxide solution at room temperature to give a dark red solution after 0.5 h. To this solution ZnCl_2_.6H_2_O (0.244 g, 1 mmol) was added. After stirring the reaction mixture for 1 h, [BDA]Br_2_ (0.486 g, 1 mmol) was added to form a red solution. The reaction mixture was filtered off after 1 h, and the filtrate was then standed. The red crystals were obtained after two weeks.

### Refinement

H atoms were placed in calculated positions with C—H = 0.97 Å and O—H = 0.82 Å and included in a riding-motion approximation with *U*_iso_(H) = 1.2*U*_eq_(C) or 1.5*U*_eq_(O). The C—O distances in the major and minor components of disorder were constrined to be similar.

### Crystal data

**Table:**

(C_16_H_38_N_2_O_6_)[Zn(C_3_S_5_)_2_]	*Z* = 2
*M**_r_* = 812.51	*F*(000) = 844
Triclinic, *P*1	*D*_x_ = 1.620 Mg m^−^^3^
*a* = 9.051 (5) Å	Mo *K*α radiation, λ = 0.71073 Å
*b* = 13.142 (7) Å	Cell parameters from 6094 reflections
*c* = 15.321 (8) Å	θ = 2.5–28.4°
α = 69.803 (5)°	µ = 1.40 mm^−^^1^
β = 84.566 (5)°	*T* = 296 K
γ = 76.909 (6)°	Block, red
*V* = 1665.6 (15) Å^3^	0.21 × 0.20 × 0.20 mm

### Data collection

**Table:**

Bruker SMART APEXII CCD diffractometer	6279 reflections with *I* > 2σ(*I*)
Radiation source: fine-focus sealed tube	*R*_int_ = 0.017
φ and ω scans	θ_max_ = 28.6°, θ_min_ = 1.7°
Absorption correction: multi-scan (*SADABS*; Bruker, 2008)	*h* = −12→11
*T*_min_ = 0.757, *T*_max_ = 0.767	*k* = −17→12
11410 measured reflections	*l* = −19→20
7406 independent reflections	

### Refinement

**Table:**

Refinement on *F*^2^	4 restraints
Least-squares matrix: full	Hydrogen site location: mixed
*R*[*F*^2^ > 2σ(*F*^2^)] = 0.035	H-atom parameters constrained
*wR*(*F*^2^) = 0.098	*w* = 1/[σ^2^(*F*_o_^2^) + (0.0483*P*)^2^ + 1.1464*P*] where *P* = (*F*_o_^2^ + 2*F*_c_^2^)/3
*S* = 1.05	(Δ/σ)_max_ = 0.001
7406 reflections	Δρ_max_ = 0.89 e Å^−^^3^
401 parameters	Δρ_min_ = −0.61 e Å^−^^3^

### Special details

**Table:**

Geometry. All e.s.d.'s (except the e.s.d. in the dihedral angle between two l.s. planes) are estimated using the full covariance matrix. The cell e.s.d.'s are taken into account individually in the estimation of e.s.d.'s in distances, angles and torsion angles; correlations between e.s.d.'s in cell parameters are only used when they are defined by crystal symmetry. An approximate (isotropic) treatment of cell e.s.d.'s is used for estimating e.s.d.'s involving l.s. planes.

### Fractional atomic coordinates and isotropic or equivalent isotropic displacement parameters (Å^2^)

**Table:**

	*x*	*y*	*z*	*U*_iso_*/*U*_eq_	Occ. (<1)
Zn1	0.72260 (3)	0.31560 (2)	0.29989 (2)	0.03324 (9)	
S1	1.43528 (9)	0.07781 (8)	0.13331 (7)	0.0595 (2)	
S2	1.16079 (8)	0.26092 (7)	0.11098 (5)	0.04481 (17)	
S3	1.19712 (7)	0.07888 (6)	0.28040 (5)	0.04110 (16)	
S4	0.86527 (8)	0.38273 (6)	0.16423 (5)	0.03975 (16)	
S5	0.91180 (7)	0.16205 (5)	0.37140 (4)	0.03560 (14)	
S6	0.60063 (7)	0.43102 (5)	0.38427 (4)	0.03266 (14)	
S7	0.49964 (7)	0.27202 (5)	0.26931 (5)	0.03467 (14)	
S8	0.18379 (7)	0.36528 (5)	0.31683 (4)	0.03174 (13)	
S9	0.26376 (7)	0.50500 (5)	0.40545 (4)	0.03092 (13)	
S10	−0.06504 (7)	0.51276 (7)	0.38168 (6)	0.04485 (18)	
C1	1.2721 (3)	0.1367 (2)	0.1725 (2)	0.0407 (6)	
C2	1.0312 (3)	0.1804 (2)	0.27378 (17)	0.0298 (5)	
C3	1.0134 (3)	0.2666 (2)	0.19272 (17)	0.0316 (5)	
C4	0.3785 (3)	0.35788 (19)	0.32191 (16)	0.0270 (5)	
C5	0.4163 (3)	0.42241 (19)	0.36549 (16)	0.0274 (5)	
C6	0.1181 (3)	0.4648 (2)	0.36886 (17)	0.0304 (5)	
O1	0.8411 (3)	1.0111 (2)	0.13299 (15)	0.0738 (8)	
H1	0.9254	0.9745	0.1272	0.111*	
O2	1.1048 (2)	0.8211 (2)	0.34668 (15)	0.0574 (6)	
H2	1.0753	0.8226	0.2972	0.086*	
O3	0.3905 (2)	0.79755 (19)	0.43313 (15)	0.0518 (5)	
H3	0.3166	0.8242	0.3994	0.078*	
N1	0.7275 (2)	0.87055 (15)	0.38146 (13)	0.0259 (4)	
C15	0.5189 (3)	0.7627 (3)	0.3815 (2)	0.0452 (7)	
H15A	0.5092	0.8112	0.3172	0.054*	
H15B	0.5190	0.6884	0.3827	0.054*	
C16	0.6697 (3)	0.76267 (19)	0.41688 (18)	0.0325 (5)	
H16A	0.7460	0.7062	0.4015	0.039*	
H16B	0.6623	0.7403	0.4842	0.039*	
C17	0.5665 (3)	0.9539 (2)	0.49775 (17)	0.0374 (6)	
H17A	0.5393	0.8826	0.5290	0.045*	
H17B	0.6525	0.9572	0.5292	0.045*	
C18	0.6089 (3)	0.9665 (2)	0.39676 (16)	0.0303 (5)	
H18A	0.6470	1.0341	0.3688	0.036*	
H18B	0.5177	0.9748	0.3642	0.036*	
C19	0.7612 (3)	0.8966 (2)	0.27804 (16)	0.0332 (5)	
H19A	0.6700	0.8987	0.2484	0.040*	
H19B	0.8382	0.8360	0.2695	0.040*	
C20	0.8147 (4)	1.0040 (3)	0.22751 (19)	0.0460 (7)	
H20A	0.7379	1.0668	0.2322	0.055*	
H20B	0.9072	1.0040	0.2547	0.055*	
C21	0.8694 (3)	0.8543 (2)	0.43514 (17)	0.0307 (5)	
H21A	0.8970	0.9258	0.4192	0.037*	
H21B	0.8433	0.8310	0.5010	0.037*	
C22	1.0087 (3)	0.7720 (2)	0.42009 (19)	0.0362 (5)	
H22A	1.0658	0.7383	0.4771	0.043*	
H22B	0.9762	0.7137	0.4066	0.043*	
C12	0.6135 (5)	0.6888 (6)	0.1276 (3)	0.0924 (16)	
H12A	0.6734	0.6384	0.0978	0.111*	0.701 (9)
H12B	0.5920	0.7623	0.0814	0.111*	0.701 (9)
O4	0.6950 (5)	0.6901 (5)	0.1991 (3)	0.096 (2)	0.701 (9)
H4	0.7745	0.7024	0.1711	0.144*	0.701 (9)
C14	0.2392 (5)	0.8699 (3)	0.1175 (3)	0.0687 (10)	
H14A	0.3198	0.8512	0.1613	0.082*	0.671 (8)
H14B	0.2246	0.9487	0.0831	0.082*	0.671 (8)
O5	0.1091 (4)	0.8486 (4)	0.1657 (3)	0.0746 (16)	0.671 (8)
H5	0.0669	0.8126	0.1455	0.112*	0.671 (8)
C7	0.0785 (5)	0.6446 (4)	0.0908 (3)	0.0781 (12)	
H7A	0.1068	0.6577	0.0258	0.094*	0.566 (7)
H7B	0.0342	0.5793	0.1131	0.094*	0.566 (7)
O6	−0.0266 (7)	0.7353 (6)	0.1000 (6)	0.132 (3)	0.566 (7)
H6	−0.0577	0.7765	0.0485	0.198*	0.566 (7)
H12C	0.6957	0.6376	0.1665	0.111*	0.299 (9)
H12D	0.6327	0.6850	0.0653	0.111*	0.299 (9)
O4'	0.6138 (17)	0.7934 (8)	0.1249 (11)	0.130 (7)	0.299 (9)
H4'	0.6945	0.8099	0.1022	0.195*	0.299 (9)
H14C	0.1848	0.8266	0.1695	0.082*	0.329 (8)
H14D	0.3292	0.8784	0.1418	0.082*	0.329 (8)
O5'	0.1495 (14)	0.9722 (7)	0.0798 (9)	0.141 (6)	0.329 (8)
H5'	0.1913	1.0077	0.0330	0.212*	0.329 (8)
H7'1	0.0479	0.7232	0.0571	0.094*	0.434 (7)
H7'2	0.1053	0.6070	0.0453	0.094*	0.434 (7)
O6'	−0.0404 (6)	0.6090 (5)	0.1422 (5)	0.074 (2)	0.434 (7)
H6'	−0.0684	0.6440	0.1781	0.111*	0.434 (7)
N2	0.3447 (3)	0.68177 (19)	0.09357 (14)	0.0369 (5)	
C8	0.2173 (3)	0.6257 (3)	0.1464 (2)	0.0472 (7)	
H8A	0.2575	0.5466	0.1713	0.057*	
H8B	0.1861	0.6516	0.1987	0.057*	
C9	0.4633 (4)	0.5148 (3)	0.0416 (2)	0.0559 (8)	
H9A	0.5367	0.4913	0.0905	0.067*	
H9B	0.3801	0.4769	0.0654	0.067*	
C10	0.4042 (3)	0.6398 (2)	0.01308 (18)	0.0424 (6)	
H10A	0.3232	0.6607	−0.0309	0.051*	
H10B	0.4854	0.6765	−0.0187	0.051*	
C11	0.4673 (3)	0.6526 (3)	0.1644 (2)	0.0528 (8)	
H11A	0.4268	0.6852	0.2118	0.063*	
H11B	0.4898	0.5728	0.1943	0.063*	
C13	0.2875 (4)	0.8067 (2)	0.0507 (2)	0.0485 (7)	
H13A	0.3672	0.8375	0.0103	0.058*	
H13B	0.2020	0.8194	0.0119	0.058*	

### Atomic displacement parameters (Å^2^)

**Table:**

	*U*^11^	*U*^22^	*U*^33^	*U*^12^	*U*^13^	*U*^23^
Zn1	0.02578 (15)	0.03610 (17)	0.03580 (16)	−0.00251 (11)	0.00140 (11)	−0.01242 (13)
S1	0.0344 (4)	0.0688 (5)	0.0720 (5)	−0.0009 (4)	0.0130 (4)	−0.0290 (4)
S2	0.0335 (4)	0.0567 (4)	0.0376 (3)	−0.0042 (3)	0.0051 (3)	−0.0122 (3)
S3	0.0283 (3)	0.0377 (3)	0.0515 (4)	−0.0017 (3)	0.0002 (3)	−0.0112 (3)
S4	0.0327 (3)	0.0404 (4)	0.0361 (3)	−0.0009 (3)	0.0001 (3)	−0.0047 (3)
S5	0.0329 (3)	0.0352 (3)	0.0331 (3)	−0.0036 (2)	0.0006 (2)	−0.0070 (3)
S6	0.0228 (3)	0.0394 (3)	0.0415 (3)	−0.0072 (2)	−0.0022 (2)	−0.0198 (3)
S7	0.0306 (3)	0.0353 (3)	0.0433 (3)	−0.0053 (2)	0.0013 (2)	−0.0211 (3)
S8	0.0252 (3)	0.0344 (3)	0.0413 (3)	−0.0095 (2)	−0.0010 (2)	−0.0175 (3)
S9	0.0248 (3)	0.0339 (3)	0.0393 (3)	−0.0058 (2)	0.0003 (2)	−0.0191 (3)
S10	0.0227 (3)	0.0550 (4)	0.0645 (5)	−0.0040 (3)	0.0011 (3)	−0.0325 (4)
C1	0.0292 (13)	0.0487 (16)	0.0478 (15)	−0.0086 (11)	0.0008 (11)	−0.0209 (13)
C2	0.0227 (11)	0.0323 (12)	0.0376 (12)	−0.0060 (9)	−0.0006 (9)	−0.0155 (10)
C3	0.0250 (12)	0.0392 (13)	0.0336 (12)	−0.0071 (10)	−0.0013 (9)	−0.0154 (11)
C4	0.0243 (11)	0.0270 (11)	0.0302 (11)	−0.0069 (8)	0.0000 (9)	−0.0092 (9)
C5	0.0221 (11)	0.0291 (11)	0.0314 (11)	−0.0057 (8)	0.0015 (9)	−0.0108 (9)
C6	0.0245 (11)	0.0317 (12)	0.0356 (12)	−0.0057 (9)	0.0005 (9)	−0.0125 (10)
O1	0.0740 (18)	0.0860 (19)	0.0353 (11)	0.0018 (14)	0.0146 (11)	−0.0034 (11)
O2	0.0394 (12)	0.0719 (15)	0.0470 (12)	−0.0044 (10)	0.0077 (9)	−0.0093 (11)
O3	0.0262 (10)	0.0638 (14)	0.0569 (13)	−0.0061 (9)	0.0003 (9)	−0.0119 (11)
N1	0.0251 (9)	0.0245 (9)	0.0274 (9)	−0.0024 (7)	0.0012 (7)	−0.0098 (8)
C15	0.0380 (15)	0.0541 (17)	0.0509 (16)	−0.0195 (13)	0.0036 (12)	−0.0216 (14)
C16	0.0303 (12)	0.0256 (11)	0.0416 (13)	−0.0056 (9)	0.0032 (10)	−0.0123 (10)
C17	0.0383 (14)	0.0381 (14)	0.0314 (12)	0.0051 (11)	0.0017 (10)	−0.0149 (11)
C18	0.0304 (12)	0.0279 (11)	0.0297 (11)	0.0035 (9)	0.0005 (9)	−0.0124 (10)
C19	0.0318 (13)	0.0392 (13)	0.0294 (12)	−0.0043 (10)	0.0028 (9)	−0.0154 (10)
C20	0.0457 (16)	0.0521 (17)	0.0334 (13)	−0.0116 (13)	0.0064 (12)	−0.0067 (12)
C21	0.0285 (12)	0.0314 (12)	0.0326 (12)	−0.0051 (9)	−0.0015 (9)	−0.0117 (10)
C22	0.0276 (12)	0.0344 (13)	0.0438 (14)	−0.0025 (10)	−0.0024 (10)	−0.0116 (11)
C12	0.052 (2)	0.166 (5)	0.070 (3)	−0.045 (3)	−0.0022 (19)	−0.038 (3)
O4	0.063 (3)	0.174 (6)	0.073 (3)	−0.052 (3)	0.000 (2)	−0.052 (3)
C14	0.074 (3)	0.055 (2)	0.081 (3)	−0.0139 (18)	0.016 (2)	−0.032 (2)
O5	0.064 (3)	0.090 (3)	0.081 (3)	−0.011 (2)	0.019 (2)	−0.051 (3)
C7	0.050 (2)	0.100 (3)	0.085 (3)	−0.029 (2)	−0.008 (2)	−0.022 (2)
O6	0.063 (4)	0.155 (7)	0.160 (7)	−0.022 (4)	−0.014 (4)	−0.027 (6)
C12'	0.052 (2)	0.166 (5)	0.070 (3)	−0.045 (3)	−0.0022 (19)	−0.038 (3)
O4'	0.118 (12)	0.135 (14)	0.144 (14)	−0.061 (10)	0.011 (10)	−0.038 (11)
C14'	0.074 (3)	0.055 (2)	0.081 (3)	−0.0139 (18)	0.016 (2)	−0.032 (2)
O5'	0.147 (13)	0.120 (11)	0.165 (13)	−0.029 (9)	0.023 (10)	−0.064 (10)
C7'	0.050 (2)	0.100 (3)	0.085 (3)	−0.029 (2)	−0.008 (2)	−0.022 (2)
O6'	0.054 (4)	0.075 (5)	0.092 (5)	−0.017 (3)	0.008 (3)	−0.026 (4)
N2	0.0359 (12)	0.0431 (12)	0.0289 (10)	−0.0098 (9)	−0.0011 (9)	−0.0073 (9)
C8	0.0456 (17)	0.0516 (17)	0.0398 (15)	−0.0170 (13)	0.0037 (12)	−0.0064 (13)
C9	0.065 (2)	0.0458 (17)	0.0473 (17)	0.0007 (15)	−0.0007 (15)	−0.0111 (14)
C10	0.0466 (16)	0.0435 (15)	0.0310 (13)	−0.0018 (12)	−0.0026 (11)	−0.0092 (11)
C11	0.0431 (17)	0.082 (2)	0.0343 (14)	−0.0149 (16)	−0.0041 (12)	−0.0180 (15)
C13	0.0537 (18)	0.0422 (16)	0.0448 (15)	−0.0095 (13)	0.0081 (13)	−0.0111 (13)

### Geometric parameters (Å, º)

**Table:**

Zn1—S5	2.3384 (11)	C19—H19B	0.9700
Zn1—S4	2.3467 (11)	C20—H20A	0.9700
Zn1—S7	2.3468 (13)	C20—H20B	0.9700
Zn1—S6	2.3487 (10)	C21—C22	1.520 (3)
S1—C1	1.666 (3)	C21—H21A	0.9700
S2—C1	1.715 (3)	C21—H21B	0.9700
S2—C3	1.748 (3)	C22—H22A	0.9700
S3—C1	1.710 (3)	C22—H22B	0.9700
S3—C2	1.753 (3)	C12—O4	1.384 (5)
S4—C3	1.744 (3)	C12—C11	1.501 (5)
S5—C2	1.740 (3)	C12—H12A	0.9700
S6—C5	1.754 (3)	C12—H12B	0.9700
S7—C4	1.739 (2)	O4—H4	0.8200
S8—C6	1.726 (3)	C14—O5	1.360 (5)
S8—C4	1.751 (3)	C14—C13	1.506 (5)
S9—C6	1.730 (3)	C14—H14A	0.9700
S9—C5	1.755 (2)	C14—H14B	0.9700
S10—C6	1.653 (3)	O5—H5	0.8200
C2—C3	1.355 (4)	C7—O6	1.387 (6)
C4—C5	1.359 (3)	C7—C8	1.510 (5)
O1—C20	1.419 (4)	C7—H7A	0.9700
O1—H1	0.8200	C7—H7B	0.9700
O2—C22	1.416 (3)	O6—H6	0.8200
O2—H2	0.8200	O4'—H4'	0.8200
O3—C15	1.422 (4)	O5'—H5'	0.8200
O3—H3	0.8200	O6'—H6'	0.8200
N1—C19	1.517 (3)	N2—C11	1.517 (4)
N1—C21	1.525 (3)	N2—C8	1.524 (4)
N1—C16	1.526 (3)	N2—C10	1.525 (3)
N1—C18	1.529 (3)	N2—C13	1.526 (4)
C15—C16	1.515 (4)	C8—H8A	0.9700
C15—H15A	0.9700	C8—H8B	0.9700
C15—H15B	0.9700	C9—C9^ii^	1.511 (6)
C16—H16A	0.9700	C9—C10	1.524 (4)
C16—H16B	0.9700	C9—H9A	0.9700
C17—C17^i^	1.517 (5)	C9—H9B	0.9700
C17—C18	1.520 (3)	C10—H10A	0.9700
C17—H17A	0.9700	C10—H10B	0.9700
C17—H17B	0.9700	C11—H11A	0.9700
C18—H18A	0.9700	C11—H11B	0.9700
C18—H18B	0.9700	C13—H13A	0.9700
C19—C20	1.520 (4)	C13—H13B	0.9700
C19—H19A	0.9700		
			
S5—Zn1—S4	95.39 (4)	C19—C20—H20A	110.3
S5—Zn1—S7	114.65 (5)	O1—C20—H20B	110.3
S4—Zn1—S7	112.42 (4)	C19—C20—H20B	110.3
S5—Zn1—S6	119.13 (4)	H20A—C20—H20B	108.5
S4—Zn1—S6	121.38 (4)	C22—C21—N1	117.3 (2)
S7—Zn1—S6	95.16 (4)	C22—C21—H21A	108.0
C1—S2—C3	98.74 (13)	N1—C21—H21A	108.0
C1—S3—C2	98.65 (13)	C22—C21—H21B	108.0
C3—S4—Zn1	94.44 (9)	N1—C21—H21B	108.0
C2—S5—Zn1	94.63 (9)	H21A—C21—H21B	107.2
C5—S6—Zn1	95.26 (8)	O2—C22—C21	113.1 (2)
C4—S7—Zn1	95.17 (9)	O2—C22—H22A	109.0
C6—S8—C4	98.40 (11)	C21—C22—H22A	109.0
C6—S9—C5	97.96 (12)	O2—C22—H22B	109.0
S1—C1—S3	123.13 (18)	C21—C22—H22B	109.0
S1—C1—S2	124.60 (18)	H22A—C22—H22B	107.8
S3—C1—S2	112.26 (15)	O4—C12—C11	110.5 (4)
C3—C2—S5	127.41 (19)	O4—C12—H12A	109.6
C3—C2—S3	115.23 (18)	C11—C12—H12A	109.5
S5—C2—S3	117.34 (14)	O4—C12—H12B	109.6
C2—C3—S4	127.16 (19)	C11—C12—H12B	109.6
C2—C3—S2	115.10 (19)	H12A—C12—H12B	108.1
S4—C3—S2	117.70 (15)	C12—O4—H4	100.0
C5—C4—S7	127.82 (18)	O5—C14—C13	113.3 (3)
C5—C4—S8	115.32 (18)	O5—C14—H14A	108.9
S7—C4—S8	116.85 (13)	C13—C14—H14A	108.9
C4—C5—S6	126.34 (18)	O5—C14—H14B	108.9
C4—C5—S9	115.79 (18)	C13—C14—H14B	108.9
S6—C5—S9	117.87 (14)	H14A—C14—H14B	107.7
S10—C6—S8	122.11 (15)	C14—O5—H5	112.3
S10—C6—S9	125.45 (15)	O6—C7—C8	109.5 (5)
S8—C6—S9	112.43 (13)	O6—C7—H7A	109.8
C20—O1—H1	109.5	C8—C7—H7A	109.8
C22—O2—H2	109.5	O6—C7—H7B	109.8
C15—O3—H3	109.5	C8—C7—H7B	109.8
C19—N1—C21	111.68 (18)	H7A—C7—H7B	108.2
C19—N1—C16	107.84 (18)	C7—O6—H6	109.5
C21—N1—C16	108.20 (18)	C11—N2—C8	105.6 (2)
C19—N1—C18	109.04 (17)	C11—N2—C10	111.1 (2)
C21—N1—C18	108.76 (18)	C8—N2—C10	110.7 (2)
C16—N1—C18	111.34 (18)	C11—N2—C13	112.3 (2)
O3—C15—C16	114.3 (2)	C8—N2—C13	110.9 (2)
O3—C15—H15A	108.7	C10—N2—C13	106.4 (2)
C16—C15—H15A	108.7	C7—C8—N2	115.9 (3)
O3—C15—H15B	108.7	C7—C8—H8A	108.3
C16—C15—H15B	108.7	N2—C8—H8A	108.3
H15A—C15—H15B	107.6	C7—C8—H8B	108.3
C15—C16—N1	117.8 (2)	N2—C8—H8B	108.3
C15—C16—H16A	107.9	H8A—C8—H8B	107.4
N1—C16—H16A	107.9	C9^ii^—C9—C10	109.6 (3)
C15—C16—H16B	107.9	C9^ii^—C9—H9A	109.8
N1—C16—H16B	107.9	C10—C9—H9A	109.8
H16A—C16—H16B	107.2	C9^ii^—C9—H9B	109.8
C17^i^—C17—C18	109.6 (3)	C10—C9—H9B	109.8
C17^i^—C17—H17A	109.8	H9A—C9—H9B	108.2
C18—C17—H17A	109.8	C9—C10—N2	114.5 (2)
C17^i^—C17—H17B	109.8	C9—C10—H10A	108.6
C18—C17—H17B	109.8	N2—C10—H10A	108.6
H17A—C17—H17B	108.2	C9—C10—H10B	108.6
C17—C18—N1	115.26 (19)	N2—C10—H10B	108.6
C17—C18—H18A	108.5	H10A—C10—H10B	107.6
N1—C18—H18A	108.5	C12—C11—N2	116.3 (3)
C17—C18—H18B	108.5	C12—C11—H11A	108.2
N1—C18—H18B	108.5	N2—C11—H11A	108.2
H18A—C18—H18B	107.5	C12—C11—H11B	108.2
N1—C19—C20	116.6 (2)	N2—C11—H11B	108.2
N1—C19—H19A	108.1	H11A—C11—H11B	107.4
C20—C19—H19A	108.1	C14—C13—N2	116.5 (3)
N1—C19—H19B	108.1	C14—C13—H13A	108.2
C20—C19—H19B	108.1	N2—C13—H13A	108.2
H19A—C19—H19B	107.3	C14—C13—H13B	108.2
O1—C20—C19	107.3 (3)	N2—C13—H13B	108.2
O1—C20—H20A	110.3	H13A—C13—H13B	107.3
			
C2—S3—C1—S1	−179.27 (18)	O3—C15—C16—N1	−86.4 (3)
C2—S3—C1—S2	1.05 (18)	C19—N1—C16—C15	−65.9 (3)
C3—S2—C1—S1	178.89 (19)	C21—N1—C16—C15	173.2 (2)
C3—S2—C1—S3	−1.43 (18)	C18—N1—C16—C15	53.7 (3)
Zn1—S5—C2—C3	7.0 (2)	C17^i^—C17—C18—N1	−172.2 (3)
Zn1—S5—C2—S3	−175.04 (12)	C19—N1—C18—C17	−178.3 (2)
C1—S3—C2—C3	−0.1 (2)	C21—N1—C18—C17	−56.3 (3)
C1—S3—C2—S5	−178.35 (15)	C16—N1—C18—C17	62.8 (3)
S5—C2—C3—S4	−0.1 (4)	C21—N1—C19—C20	−63.6 (3)
S3—C2—C3—S4	−178.16 (14)	C16—N1—C19—C20	177.6 (2)
S5—C2—C3—S2	177.16 (14)	C18—N1—C19—C20	56.6 (3)
S3—C2—C3—S2	−0.9 (3)	N1—C19—C20—O1	179.3 (2)
Zn1—S4—C3—C2	−6.8 (2)	C19—N1—C21—C22	−52.4 (3)
Zn1—S4—C3—S2	175.99 (13)	C16—N1—C21—C22	66.2 (3)
C1—S2—C3—C2	1.4 (2)	C18—N1—C21—C22	−172.8 (2)
C1—S2—C3—S4	178.98 (15)	N1—C21—C22—O2	90.2 (3)
Zn1—S7—C4—C5	−1.2 (2)	O6—C7—C8—N2	−92.6 (5)
Zn1—S7—C4—S8	177.39 (12)	C11—N2—C8—C7	178.7 (3)
C6—S8—C4—C5	3.0 (2)	C10—N2—C8—C7	−61.0 (4)
C6—S8—C4—S7	−175.81 (14)	C13—N2—C8—C7	56.8 (4)
S7—C4—C5—S6	−2.9 (3)	C9^ii^—C9—C10—N2	−172.9 (3)
S8—C4—C5—S6	178.43 (13)	C11—N2—C10—C9	59.0 (3)
S7—C4—C5—S9	176.60 (14)	C8—N2—C10—C9	−57.9 (3)
S8—C4—C5—S9	−2.0 (3)	C13—N2—C10—C9	−178.5 (3)
Zn1—S6—C5—C4	5.0 (2)	O4—C12—C11—N2	160.9 (4)
Zn1—S6—C5—S9	−174.57 (12)	C8—N2—C11—C12	174.2 (4)
C6—S9—C5—C4	0.1 (2)	C10—N2—C11—C12	54.1 (4)
C6—S9—C5—S6	179.63 (14)	C13—N2—C11—C12	−64.9 (4)
C4—S8—C6—S10	177.80 (16)	O5—C14—C13—N2	−69.8 (5)
C4—S8—C6—S9	−2.82 (16)	C11—N2—C13—C14	−51.4 (4)
C5—S9—C6—S10	−178.71 (17)	C8—N2—C13—C14	66.4 (4)
C5—S9—C6—S8	1.93 (16)	C10—N2—C13—C14	−173.1 (3)

Symmetry codes: (i) −*x*+1, −*y*+2, −*z*+1; (ii) −*x*+1, −*y*+1, −*z*.

### Hydrogen-bond geometry (Å, º)

**Table:**

*D*—H···*A*	*D*—H	H···*A*	*D*···*A*	*D*—H···*A*
O1—H1···O5^iii^	0.82	2.03	2.800 (5)	157
O2—H2···O5^iii^	0.82	1.93	2.668 (5)	149
O3—H3···O2^iv^	0.82	2.16	2.923 (3)	154
O4—H4···O6^iii^	0.82	2.07	2.887 (8)	179
O5—H5···O6	0.82	1.79	2.615 (9)	179
O6—H6···S2^ii^	0.82	2.92	3.540 (8)	134

Symmetry codes: (ii) −*x*+1, −*y*+1, −*z*; (iii) *x*+1, *y*, *z*; (iv) *x*−1, *y*, *z*.
